# Sustainable Development of PLA-Based Biocomposites Reinforced with Pineapple Core Powder: Extrusion and 3D Printing for Thermal and Mechanical Performance

**DOI:** 10.3390/polym17131792

**Published:** 2025-06-27

**Authors:** Kawita Chattrakul, Anothai Pholsuwan, Athapon Simpraditpan, Ekkachai Martwong, Wichain Chailad

**Affiliations:** 1 Department of Materials and Metallurgical Engineering, Faculty of Engineering, Rajamangala University of Technology Thanyaburi, Pathum Thani 12110, Thailand; kawita.c@en.rmutt.ac.th (K.C.); anothai.p@en.rmutt.ac.th (A.P.); athapon.s@en.rmutt.ac.th (A.S.); 2 Division of Science, Faculty of Science and Technology, Rajamangala University of Technology Suvarnabhumi, Phra Nakhon Si Ayutthaya 13000, Thailand

**Keywords:** PLA biocomposites, pineapple core powder, surface modification, 3D printing, mechanical properties

## Abstract

This study developed sustainable biocomposites composed of polylactic acid (PLA) and surface-treated pineapple core powder (PACP), fabricated via extrusion and fused deposition modelling (FDM). PACP loadings of 1–3 vol% were combined after chemical modification with NaOH and silane to improve interfacial bonding. Particle morphology showed increased porosity and surface roughness following treatment. The melt flow index (MFI) increased from 31.56 to 35.59 g/10 min at 2 vol% PACP, showing improved flowability. Differential scanning calorimetry (DSC) showed the emergence of cold crystallization (T_cc_ ~121 °C) and an increase in crystallinity from 35.7% (neat PLA) to 47.3% (2 vol% PACP). Thermogravimetric analysis showed only slight decreases in T_5_ and T_max_, showing the thermal stability. The mechanical testing of extruded filaments showed increased modulus (1463 to 1518 MPa) but a decrease in tensile strength and elongation. For the 3D-printed samples, elongation at break increased slightly at 1–2 vol% PACP, likely because of the improvement in interlayer fusion. Though, at 3 vol% PACP, the mechanical properties declined, consistent with filler agglomeration observed in SEM. Overall, 2 vol% PACP offered the optimal balance between printability, crystallinity, and mechanical performance. These results reveal the possibility of PACP as a value-added biowaste filler for eco-friendly PLA composites suitable for extrusion and 3D printing applications.

## 1. Introduction

Polylactic acid (PLA) is a biodegradable thermoplastic synthesised from renewable resources such as corn, cassava, and sugarcane [1,2]. Recognised for its environmental friendliness and compostability, PLA has emerged as a promising alternative to petroleum-based polymers [3,4]. Despite these advantages, PLA suffers from several intrinsic limitations—namely, brittleness, low impact resistance, poor thermal stability, and limited processability—that hinder its use in demanding or structural applications [5,6,7]. To address these shortcomings, researchers have increasingly explored the modification of PLA through the incorporation of natural fillers derived from agricultural residues. These bio-based additives can improve melt flow, enhance mechanical properties, reduce production costs, lower energy consumption during processing, and expand PLA’s potential in areas such as additive manufacturing and consumer goods [8,9,10].

Recent developments have increasingly focused on reinforcing PLA with waste-derived fillers to promote sustainable material design. For example, rice husk [11], coffee grounds [12], banana peel [13], and oil palm biomass microcrystalline cellulose [14] have been successfully integrated into PLA matrices to improve mechanical properties and reduce environmental impact. These studies demonstrate the feasibility of using agricultural by-products as low-cost reinforcements for bio-based polymers, supporting the valorisation of pineapple core powder in the present work.

Natural fibre-reinforced biocomposites offer an environmentally sustainable alternative to synthetic fibre composites. These materials typically consist of natural fibres embedded in a polymer matrix, along with compatibilisers or plasticisers to improve interfacial bonding. When used in PLA matrices, natural fibres confer the benefits of low density, high specific strength and stiffness, biodegradability, and reduced environmental impact [15,16,17]. Commonly studied fibres include hemp, jute, flax, and pineapple leaf fibre (PALF), all of which have been applied in packaging, automotive parts, household goods, helmets, and construction materials [18,19,20]. Natural fibres are primarily composed of cellulose, hemicellulose, and lignin, which together contribute to their reinforcing ability. However, challenges remain: These fibres are hydrophilic, thermally unstable, and exhibit variability in structure and properties due to differences in their botanical origin and growing conditions. These factors complicate their integration into hydrophobic polymers like PLA, making appropriate processing conditions and surface treatments essential [21,22,23].

Among these fibres, PALF has received considerable attention due to its high cellulose content (up to 82%), high tensile strength (180–1627 MPa), and Young’s modulus typically ranging from 34.5 to 82.5 GPa, depending on the extraction method and testing conditions. The higher stiffness and strength support its suitability as a reinforcement material for structural and semi-structural applications [21,22,23]. Table 1 summarises the key properties of PALF. In Thailand, where pineapple cultivation is widespread—particularly in Prachuap Khiri Khan Province, which yields over 414,000 tonnes annually across more than 400 km^2^ of farmland—pineapple leaf waste is typically discarded or incinerated, contributing to environmental burden and resource inefficiency [24]. Valorisation of this biomass into composites offers a sustainable waste management solution.

Nonetheless, the hydrophilicity of PALF leads to poor adhesion with hydrophobic polymers, such as PLA, thereby reducing composite performance [25,26]. Chemical modifications, including alkali treatment with sodium hydroxide (NaOH) and surface functionalisation using silane coupling agents such as 3-(triethoxysilyl)propyl tetrasulfide and 3-(trimethoxysilyl) propyl methacrylate, have been shown to significantly improve fibre–matrix interactions [27,28,29]. Moreover, the hybridisation of PALF with other natural fibres has yielded notable improvements in tensile, compressive, and impact properties [30,31,32].

**Table 1:** Components and selected properties of pineapple leaf fibres (PALF) [33].

Components and Properties	PALF
Ash content (%)	1.1%
Lignin content (%)	5–12%
Cellulose content (%)	70–82%
Density (g/cm^3^)	1.53
Tensile modulus (GPa)	5.83

While most studies have focused on pineapple leaf fibres [19,26,33,34], less attention has been given to the pineapple core, a by-product of fruit processing that is also rich in cellulose. Typically discarded or composted, the pineapple core offers similar compositional advantages and is more readily available as a uniform powder [35]. Its potential as a reinforcement for biocomposites remains largely unexplored, despite its suitability for applications requiring low-cost, sustainable materials. Utilising pineapple core powder (PACP) presents a unique opportunity to upcycle food processing waste into functional biocomposites. In addition to reducing agricultural waste, PACP-filled PLA composites can be utilised in various practical applications, including biodegradable packaging, disposable utensils, lightweight consumer products, and educational or prototyping components produced via 3D printing.

Among various additive manufacturing techniques, fused deposition modelling (FDM) has gained wide acceptance for fabricating thermoplastic-based composites due to its cost-effectiveness, design flexibility, and ease of use. Compared to stereolithography (SLA), which relies on UV-curable resins with brittle characteristics and is more suitable for precision optics or lighting applications [36], or selective laser melting (SLM), which is tailored for metallic powders, FDM is better suited for processing bio-based thermoplastics like PLA. Its ability to produce complex geometries at low cost and with minimal material waste makes it a preferred platform for research in sustainable polymer composites, especially those involving natural filler reinforcements [37,38].

This study investigates the fabrication and characterisation of PLA-based biocomposites reinforced with PACP at varying filler contents. The materials were produced using two fabrication techniques: traditional extrusion and fused deposition modelling (FDM), a widely used 3D printing method. The research examines how the incorporation of PACP influences melt flow behaviour, mechanical performance, morphology, and thermal stability. Ultimately, this work aims to demonstrate the viability of PACP as a reinforcing filler and contribute to the development of sustainable materials aligned with circular economy principles. By turning waste into value-added composites, this research supports resource efficiency, material innovation, and environmentally responsible manufacturing.

## 2. Materials and Methods

### 2.1. Materials and Sample Preparation

**Figure 1:**
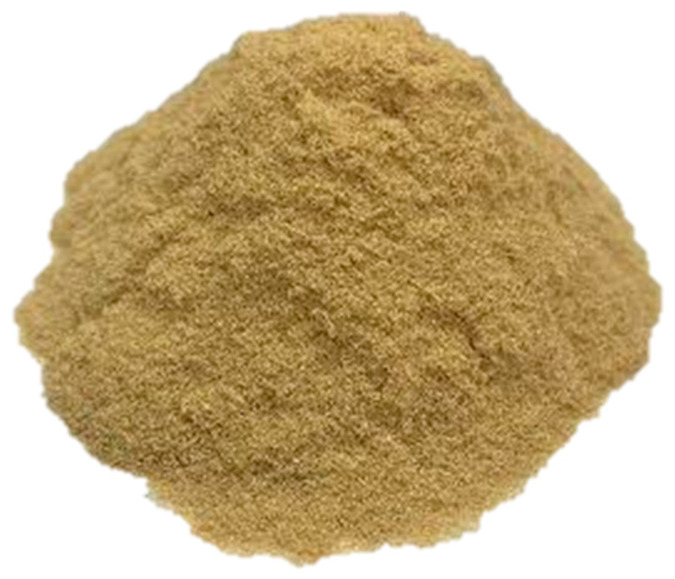
Pineapple core powder (PACP), derived from Pattavia variety pineapples grown in Thailand.

**Figure 2:**
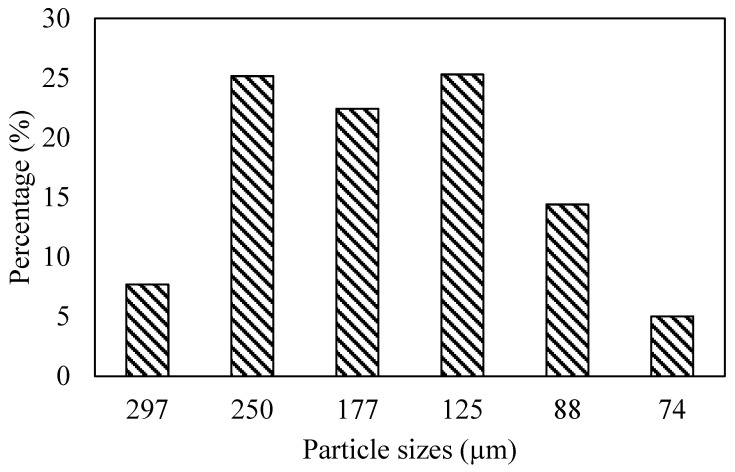
Particle size distribution of silane-treated pineapple core powder (PACP).

Polylactic acid (PLA) pellets (grade 2003D) were supplied by NatureWorks LLC (Plymouth, MN, USA). Pineapple core powder (PACP) (Figure 1), derived from the Pattavia variety of pineapples grown in Thailand, was obtained from Kongkiat Textile Co., Ltd. (Saraburi, Thailand). The pineapple cores were first cleaned to remove residual sugars and fibrous outer material, sliced, and then oven-dried at 60 °C for 24 h to reduce the moisture content. The dried cores were ground using a high-speed grinder and sieved through an 80-mesh screen to obtain a uniform fine powder. The average particle size was measured to be 172.15 ± 68.98 µm (Figure 2), and the powder was stored in a desiccator prior to use.

To enhance compatibility with the PLA matrix, the PACP underwent surface modification. First, the powder was treated with a 10 wt% sodium hydroxide (NaOH, Sigma-Aldrich, St. Louis, MO, USA) solution at room temperature for 1 h to remove lignin and hemicellulose, thereby increasing the exposure of hydroxyl groups on the fibre surface. The treated powder was neutralised using a 2 wt% acetic acid (CH_3_COOH, 99.5%, Sigma-Aldrich) solution, filtered, and initially air-dried at room temperature for 24 h, followed by oven drying at 80 °C for another 24 h. Subsequently, the PACP was functionalised with 3 wt% 3-(trimethoxysilyl)propyl methacrylate (97%, Sigma-Aldrich) to improve interfacial adhesion with the PLA. The silane-coated powder was filtered and air-dried for 12 h, followed by curing at 100 °C. The moisture content of the final surface-modified PACP was maintained below 2%.

**Figure 3:**
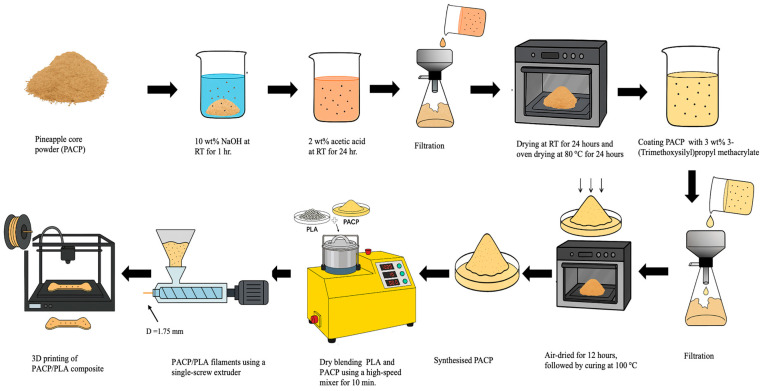
Schematic diagram of the preparation and processing of PLA and PLA/PACP biocomposites and 3D printing.

PLA and PACP were dry-blended at filler contents of 0, 1, 2, and 3 vol% using a high-speed mixer (Labtech High Speed Mixers, Labtech Engineering Co., Ltd., Samut Prakan, Thailand) for 10 min. The mixtures were then extruded into filaments using a single-screw extruder. The extrusion parameters are summarised in Table 2. The resulting filaments had a diameter of 1.75 ± 0.1 mm and were used as feedstock for 3D printing. The entire preparation process is illustrated in Figure 3.

### 2.2. Three-Dimensional Printing of PACP/PLA Composites

**Figure 4:**
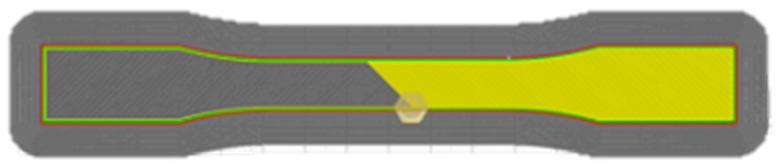
Infill pattern used for 3D-printed samples (Line +45°/−45°).

The extruded composite filaments were processed using an ANET ET5 fused deposition modelling (FDM) 3D printer. The printing conditions are listed in Table 3, and the infill pattern used is illustrated in Figure 4. These parameters were selected based on standard PLA printing guidelines and prior optimisation trials to ensure dimensional consistency and layer fusion quality. A nozzle temperature of 205 °C was used to promote proper flow of the PLA matrix without thermal degradation, while the build plate temperature of 60 °C was chosen to minimise warping and delamination. The nozzle diameter of 0.6 mm was selected to accommodate any slight filler agglomerates and maintain continuous extrusion. A 100% infill density and a bi-directional Line +45°/−45° raster pattern were employed to standardise mechanical testing and reduce anisotropy across printed layers, following approaches adopted in similar studies of natural fibre-reinforced PLA composites [39,40,41].

### 2.3. Characterisation

#### 2.3.1. Morphological Analysis

The morphology of both untreated and surface-treated pineapple core powder (PACP), along with the fracture surfaces of PLA/PACP composite specimens, was examined using a scanning electron microscope (SEM; model JSM-IT210, JEOL Ltd. Tokyo, Japan). The analysis was performed under high vacuum with an accelerating voltage of 15 kV. Before imaging, all samples were sputter-coated with a thin layer of gold to improve surface conductivity and image resolution.

#### 2.3.2. Melt Flow Index (MFI)

The melt flow index was measured using an INSTRON melt flow tester in accordance with ASTM D1238 [42]. The test was performed at 210 °C under a 2.16 kg load. The MFI was reported as the mass of material extruded through the die in 10 min (g/10 min).

#### 2.3.3. Thermal Characterisation

Thermogravimetric analysis (TGA) was carried out with a METTLER TOLEDO TGA/DSC3+ following ASTM E2550 [43] to study the thermal stability and degradation behaviour. Samples were heated from 30 to 600 °C at 10 °C/min under nitrogen flow.

Differential scanning calorimetry (DSC) was performed according to ASTM D3418 [44] using a METTLER TOLEDO DSC3+ to determine the glass transition temperature (T_g_), cold crystallisation temperature (T_c_), melting temperature (T_m_), and crystallinity (X_c_). The test range was 30–250 °C at a heating rate of 10 °C/min under a nitrogen atmosphere.

#### 2.3.4. Mechanical Testing

The tensile properties of the extruded filaments were evaluated using a universal testing machine (Model H50KS) in accordance with ASTM D2256 [45]. The crosshead speed was set at 300 mm/min with a gauge length of 250 mm.

**Figure 5:**
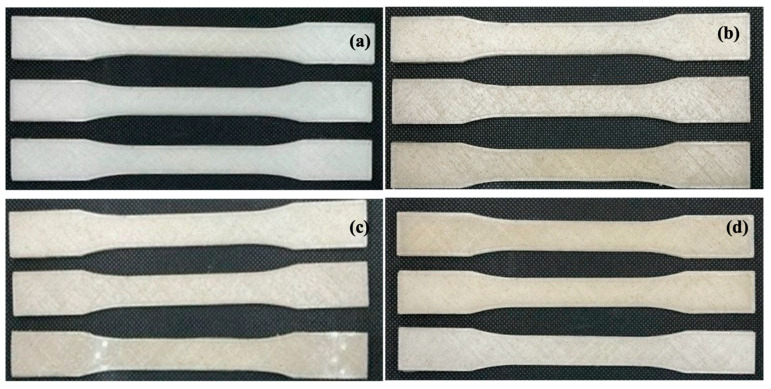
Three-dimensional-printed tensile specimens of PLA/PACP composites with 0 (**a**), 1 (**b**), 2 (**c**), and 3 (**d**) vol% PACP. Each set consists of multiple replicates fabricated using identical FDM printing conditions. Due to the low filler content and good dispersion, visual differences between the samples are not prominent. Material performance differences are instead reflected in mechanical testing and fracture surface morphology.

Tensile testing of 3D-printed specimens was carried out following ASTM D638 Type I standards. Samples were printed with a gauge length of 115 mm, a thickness of 3 mm, and tested at a speed of 50 mm/min. Representative tensile specimens printed with varying PACP contents (0, 1, 2, and 3 vol%) are shown in Figure 5. Although no prominent visual differences are apparent among the samples, this is expected due to the low filler contents (1–3 vol%) and the relatively uniform dispersion of PACP within the PLA matrix. The primary effects of PACP addition are reflected in the mechanical properties and fracture surface morphology, as detailed in later sections.

## 3. Results

### 3.1. PACP Property

#### Morphology of PACP

**Figure 6:**
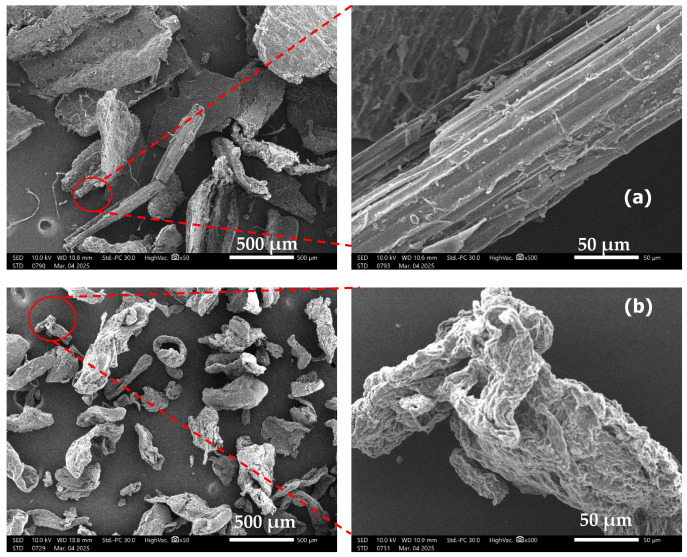
SEM images of unmodified (**a**) and modified (**b**) PACP.

The surface morphology of pineapple core powder (PACP), both untreated and chemically modified, was examined using scanning electron microscopy (SEM). As shown in Figure 6a, the unmodified PACP exhibits a wide variation in particle shape and size, ranging from flaky structures to irregular agglomerates and short fibre bundles. Many of these particles appear fused or entangled, with coarse and uneven surfaces. This surface texture is commonly associated with the presence of residual non-cellulosic constituents such as hemicellulose, lignin, and surface impurities, which form a barrier over the cellulose microfibrils and limit polymer matrix interaction [46,47,48].

After sequential alkali and silane treatment, as shown Figure 6b, the PACP particles exhibit distinctly rougher and more fibrillated and porous surfaces. The treatment results in partial defibrillation and enhanced texturing of the particle surface. This morphological change is due to the removal of amorphous components during NaOH treatment, which breaks down the lignin–hemicellulose network and exposes hydroxyl-rich cellulose surfaces. The subsequent silane functionalisation further contributes to surface etching and chemical activation, facilitating stronger interfacial bonding with PLA [49,50].

These changes in surface morphology validate the effectiveness of the chemical treatment in improving filler–matrix compatibility and support the improved mechanical behaviour and fracture morphology observed in PACP-reinforced PLA composites. The increased roughness and porosity of treated PACP promote better mechanical interlocking and stress transfer within the composite matrix.

### 3.2. PLA/ PACP Composites

#### 3.2.1. Melt Flow Index (MFI)

The melt flow index (MFI) was evaluated to investigate the effect of PACP addition on the processability of PLA. As presented in Table 4, the MFI of neat PLA was measured at 31.560 g/10 min, while the MFI values of PLA/PACP composites increased to 32.124, 35.592, and 35.004 g/10 min with PACP contents of 1, 2, and 3 vol%, respectively. The overall increasing trend in MFI indicates that the presence of PACP contributes to a reduced melt viscosity, thereby enhancing the flowability of the composite under processing conditions. This phenomenon can be attributed to several factors. First, the relatively small and rigid filler particles may disrupt the entanglement of PLA molecular chains, resulting in a reduction in resistance to shear flow. Second, PACP, particularly after surface modification, may enhance interfacial slippage between the polymer and filler phases, thereby facilitating melt flow.

The maximum improvement in MFI was observed at 2 vol% PACP, suggesting this to be an optimal loading for flow enhancement. However, at 3 vol%, a slight decrease in MFI was noted compared to the 2 vol% composite. This decline may be attributed to particle agglomeration, which leads to heterogeneity in the melt and localised obstruction to flow pathways. Non-uniform dispersion at a higher filler content could also increase interparticle friction, offsetting the shear-thinning benefits provided at lower concentrations. Despite the slight reduction at 3 vol%, the MFI remained higher than that of neat PLA, indicating that PACP, even at elevated levels, can support acceptable flow behaviour. These results suggest that PACP can be effectively used as a low-cost, biodegradable additive to improve the processability of PLA, particularly for extrusion and 3D printing applications.

#### 3.2.2. Thermal Properties

**Figure 7:**
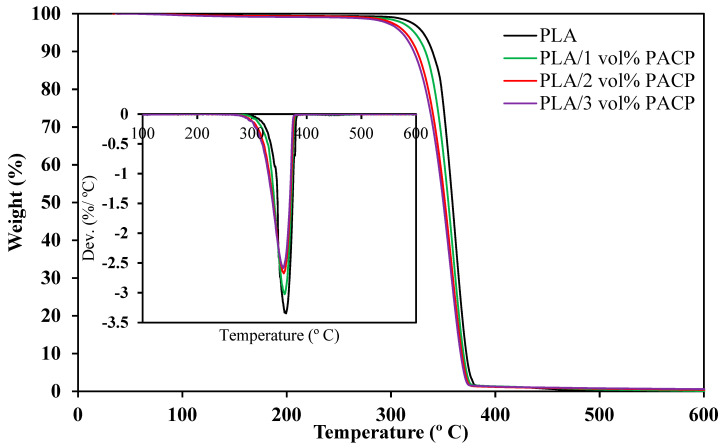
TGA results for PLA and PACP-filled PLA composites.

The thermal stability of PLA and PLA/PACP composites was assessed via thermogravimetric analysis (TGA), as shown in Figure 7 and Table 5. All samples exhibited a typical single-step degradation pattern consistent with PLA-based materials, with initial decomposition (T_5_, 5% weight loss) occurring at 327.6 °C for neat PLA. Incorporation of PACP slightly decreased the T_5_ to 322.5, 313.8, and 310.2 °C for 1, 2, and 3 vol% PACP, respectively. These small reductions are attributed to the presence of thermally unstable constituents in PACP, such as residual hemicellulose and lignin, which degrade at lower temperatures [46,47,48].

Notably, the magnitude of these shifts is minimal, and the overall degradation onset remains above 310 °C for all composites. This suggests that PACP inclusion does not significantly compromise the thermal processability of PLA for melt-based fabrication. The T_max_ (temperature at the maximum degradation rate) also decreased only slightly, from 362.5 °C in neat PLA to 358.1 °C in the 3 vol% sample, further indicating that the overall thermal degradation profile is only marginally affected.

The limited change in thermal behaviour may be attributed to the relatively low filler loadings (1–3 vol%), which were selected to preserve the mechanical integrity and printing performance. At these concentrations, PACP acts more as a passive filler than a barrier or thermal stabiliser. Unlike nanofillers or chemically modified mineral additives, PACP does not impart a significant char-forming ability or thermal shielding effects. Therefore, the results are consistent with other studies using untreated or slightly modified biomass fillers in PLA, which report modest or negligible improvements in thermal stability under low loading conditions.

Compared with the previous works by Boughanmi O et al. [12] and Komal UK et al. [13] using spend coffee ground and banana peel-reinforced PLA, the PACP-filled composites in this study exhibited similar thermal onset degradation trends, with only slight reductions in T_5_ and T_max_. This suggests that PACP behaves similarly to other cellulose-rich biowastes with minimal adverse effect on thermal stability when added at low volume fractions.

The DTG curves show a reduced degradation rate with the increasing PACP content, with the maximum weight loss rate decreasing from −3.346%/°C in neat PLA to −2.584%/°C at 3 vol% PACP. This flattening suggests a more gradual degradation, possibly due to restricted chain mobility or heterogeneous dispersion of PACP within the matrix. Additionally, char residue at 600 °C increased slightly from 0.14% to 0.59% with the rising PACP content, reflecting the higher inorganic ash content in PACP, primarily composed of thermally stable minerals such as potassium, calcium, and magnesium [51,52].

In summary, the addition of PACP at 1–3 vol% results in no significant thermal enhancement but does not deteriorate the thermal stability to a critical extent, confirming that these composites remain suitable for thermoplastic processing. The trade-off is acceptable given the bio-based origin, cost-effectiveness, and sustainability of PACP as a filler.

##### Differential Scanning Calorimetry (DSC)

**Figure 8:**
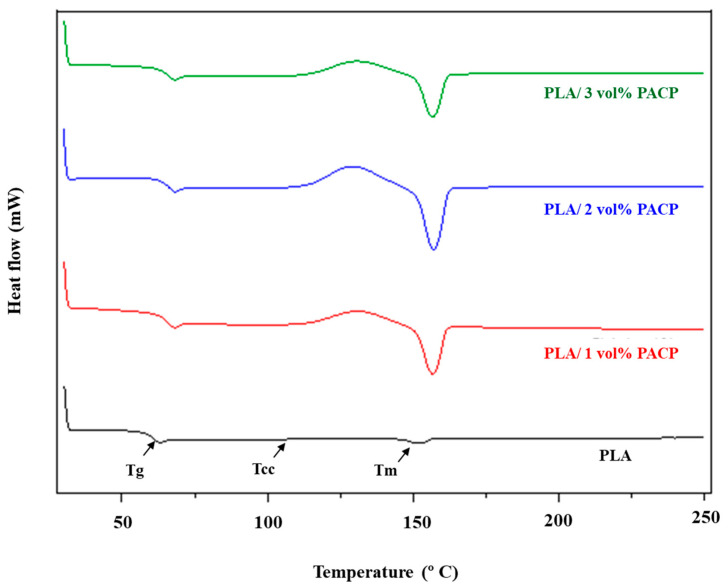
DSC results for PLA and PACP-filled PLA composites.

Differential scanning calorimetry (DSC) was performed to investigate the thermal transitions of PLA and PACP-filled PLA composites. The thermograms are shown in Figure 8, and the corresponding thermal parameters are summarised in Table 6. The thermal transition temperatures include the glass transition temperature (T_g_), cold crystallisation temperature (T_cc_), and melting temperature (T_m_), along with the degree of crystallinity (X_c_). The T_g_ of neat PLA was 59.66 °C. Upon the addition of PACP, the T_g_ slightly decreased to 57.13 °C, 59.37 °C, and 58.44 °C for the 1, 2, and 3 vol% PACP composites, respectively. This small reduction in T_g_ suggests that the filler may have interfered with the packing or cohesive interactions among the PLA chains in the amorphous regions, thereby increasing chain mobility. Such behaviour is often observed when natural fillers partially disrupt the matrix structure [53].

Cold crystallisation behaviour, reflected by the T_cc_ peak, was absent in neat PLA under the testing conditions. This implies that either PLA remained largely amorphous or the crystalline domains were too minor to detect using DSC [54,55]. In contrast, all PACP-filled composites exhibited clear T_cc_ peaks, occurring around 121 °C, indicating that PACP promotes cold crystallisation during heating. This behaviour can be attributed to the nucleating effect of PACP particles, which serve as crystallisation sites that facilitate the alignment and ordering of PLA chains into crystalline regions [56]. The observed increase in cold crystallisation (T_CC_) and crystallinity (X_C_) with PACP addition is consistent with trends reported by Kang et al. [57] for PLA composites reinforced with biomass, where biofiller acted as a nucleating agent to promote crystallisation. The increase in X_C_ values in our study (up to 57.8%) falls within the typical enhancement range reported for similar natural filler systems.

Despite changes in T_g_ and T_c_, the melting temperature (T_m_) remained relatively constant across all samples, with values ranging narrowly between 151.41 °C and 151.88 °C. This suggests that PACP incorporation did not significantly alter the crystalline phase structure or melting behaviour of PLA, which is typical for many filler–PLA systems. The degree of crystallinity (X_c_) increased noticeably with the PACP addition, especially at 1 and 2 vol% loading. The X_c_ increased from 35.71% in neat PLA to 40.27% and 47.29% in the 1 and 2 vol% PACP composites, respectively. This enhancement in crystallinity reinforces the nucleating role of PACP and is consistent with the emergence of T_cc_ peaks [58,59]. Interestingly, X_c_ dropped to 38.19% at 3 vol%, which may be due to filler agglomeration or excessive nucleation that limits effective crystal growth. Overall, these findings indicate that PACP acts as an effective nucleating agent in PLA composites. The improved crystallisation behaviour could benefit thermal resistance and dimensional stability, which are desirable in applications involving heat exposure, such as in 3D printing and hot stage processing.

#### 3.2.3. Mechanical Properties of Extruded PLA and PLA/PACP Filaments

The results of the tensile properties of extruded PLA and PLA/PACP composite filaments are summarised in Table 7. The analysis focused on three key mechanical parameters: ultimate tensile strength, Young’s modulus, and elongation at break. The addition of PACP into the PLA matrix led to a noticeable decline in ultimate tensile strength. Neat PLA exhibited the highest strength at 52.43 MPa, while the composites containing 1, 2, and 3 vol% PACP showed reduced values of 46.78, 46.91, and 45.57 MPa, respectively. This reduction suggests that the incorporation of rigid filler particles weakens the material’s ability to withstand the tensile load before failure. The filler–matrix interface may act as a stress concentrator, especially if the dispersion is uneven or interfacial bonding is limited [60,61].

In contrast, the tensile modulus demonstrated an increasing trend with PACP content, particularly at 2 and 3 vol%. The modulus increased from 1463 MPa in neat PLA to 1518 MPa at 2 vol%, indicating an enhancement in stiffness. This suggests that PACP reinforces the composite by restricting chain mobility under the tensile load. However, at 1 vol% PACP, a slight decrease in the modulus was observed (1412.50 MPa), which may imply that, at lower concentrations, the filler does not form an effective reinforcing network or is insufficiently bonded to contribute to mechanical rigidity. The tensile modulus enhancement observed at 2 vol% PACP is comparable to that reported by Wangwang et al. [62], who incorporated 3 wt% spent coffee grounds into PLA and noted a similar trade-off between stiffness and ductility. Our elongation at break values align with their findings, further supporting PACP’s suitability as a functional biofiller.

The elongation at break decreased significantly upon PACP addition, dropping from 5.58% in neat PLA to a range of 4.38–5.05% in the composites. This reduction in ductility is characteristic of particulate-filled systems and can be attributed to the presence of hard PACP particles that act as microdefect sites, impeding polymer chain movement and promoting early failure. These stress concentrators limit the material’s ability to deform plastically before fracture [60,61].

#### 3.2.4. Mechanical Properties of 3D-Printed PLA and PLA/PACP Filaments

The mechanical properties of 3D-printed PLA and PLA/PACP composites were evaluated, and the results are summarised in Table 8. Unlike the extruded filaments, the mechanical response of the printed samples revealed distinct trends influenced by both the filler content and processing methods. The ultimate tensile strength of neat PLA was 4.52 MPa. For composites with 1 and 2 vol% PACP, the values remained relatively unchanged, 4.50 and 4.65 MPa, indicating no significant compromise in strength at low filler loadings. However, a noticeable drop was observed at 3 vol%, where the strength declined to 3.82 MPa. This suggests that an excessive PACP content may introduce microstructural weaknesses in the 3D-printed part, likely due to filler agglomeration or poor interlayer fusion, thereby reducing its load-bearing capacity. This interpretation is supported by fracture morphology observations (discussed in Section 3.2.5), which show visible voids and stress concentrations in high-PACP samples. Young’s modulus followed a similar pattern. PLA printed without filler exhibited a modulus of 267 MPa, while the addition of 1 and 2 vol% PACP resulted in slight variations (253.50 and 272.67 MPa). However, a significant reduction to 223.00 MPa was observed at 3 vol%, further indicating that excessive filler may interfere with effective stress transfer between printed layers or compromise the internal structure [60,61].

In contrast to the extruded samples, where the elongation at break consistently declined with the addition of PACP, the 3D-printed specimens showed a moderate increase in ductility at 1% and 2% PACP. The elongation values increased from 2.44% in neat PLA to 2.70% and 3.00%, respectively. This improvement may indicate that, under the specific conditions of fused deposition modelling (FDM), PACP facilitates better polymer chain mobility or interlayer adhesion, possibly acting as a process-specific plasticiser or promoting melt flow during deposition. This hypothesis is supported by the earlier MFI results (Section 3.2.1), which show that PACP addition enhances flow characteristics.

The improved flow during extrusion through the nozzle may contribute to more consistent deposition and stronger bonding between printed layers. This effect could reduce porosity and enhance load distribution during deformation, resulting in better flexibility or toughness in the printed samples. However, at 3 vol% PACP, the elongation slightly dropped to 2.55%, likely due to the onset of filler agglomeration and weakening of interlayer cohesion.

While the overall mechanical performance of the PACP-filled composites did not show substantial improvement compared to neat PLA, particularly in tensile strength, this trend is consistent with findings from other studies involving low filler loadings of natural biowaste materials. The marginal enhancement or even reduction in strength is a known limitation when using unreinforced natural fillers at low concentrations without additional compatibilisers or plasticisers. Moreover, the tensile strength of neat PLA observed in this study (~4.52 MPa) appears low when compared to typical bulk PLA values (50–60 MPa). However, this discrepancy is expected due to the layer-by-layer deposition characteristic of FDM printing, which introduces interfacial weaknesses and anisotropy. Similar tensile strength values (3–6 MPa) for FDM-printed PLA parts at 100% infill have been reported by Nida Naveed. [63] and Tusharbhai et al. [64], validating that our results fall within the anticipated range for printed specimens. The data indicate that, although the PACP reinforcement does not significantly enhance the tensile strength, it slightly improves ductility under certain conditions. This suggests that PACP acts more as a flow modifier than a strength enhancer in the FDM context. Further improvements may be achieved with fibre alignment, higher filler loading, or compatibilisation strategies.

#### 3.2.5. Fracture Morphology of 3D-Print Specimens

**Figure 9:**
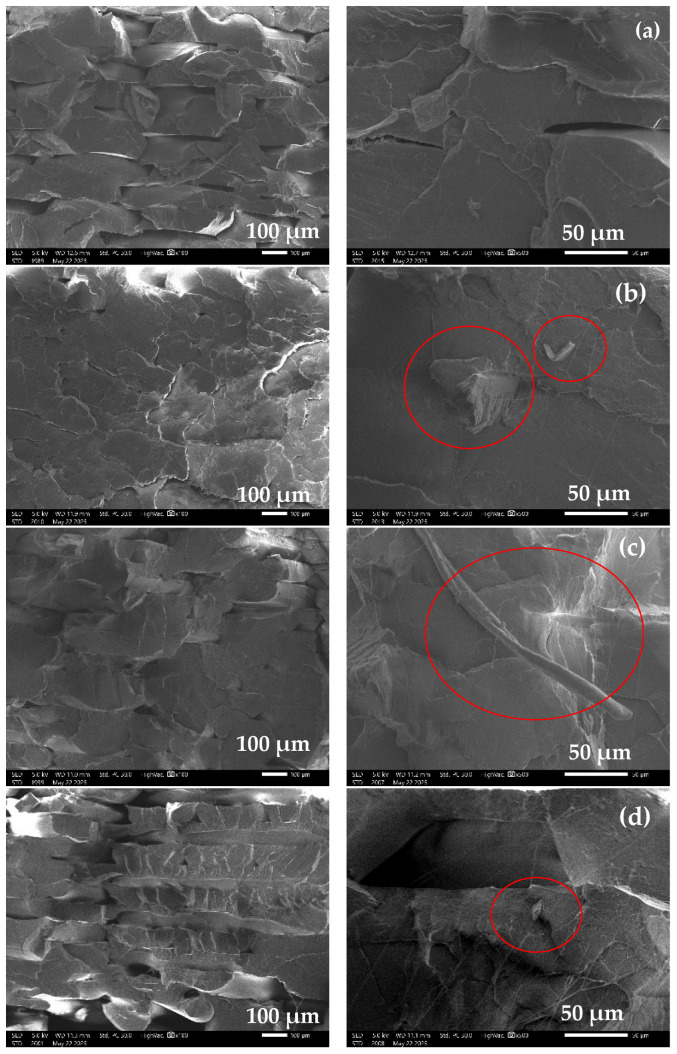
SEM images of fracture surfaces of PLA and PLA/PACP 3D-printed composites: neat PLA (**a**), PLA/1 vol% PACP (**b**), PLA/2 vol% PACP (**c**), and PLA/3 vol% PACP 9 (**d**).

The fracture surfaces of 3D-printed PLA and PLA/PACP composites were examined using SEM to study failure mechanisms and interfacial characteristics. Representative SEM micrographs are presented in Figure 9, highlighting the effects of the PACP content on filler dispersion, interlayer adhesion, and crack propagation. The fracture surface of neat PLA (Figure 9a) exhibited a relatively smooth and flat morphology, indicative of a brittle fracture. No signs of plastic deformation were observed, and the interlayer boundaries were clearly discernible. These distinct layer interfaces, a typical artifact of FDM printing, act as stress concentrators and are known contributors to reduced mechanical performance compared to conventionally moulded specimens [65].

For PLA containing 1 vol% PACP (Figure 9b), the fracture morphology remained predominantly brittle. Some dispersed PACP particles were evident; however, multiple voids and pull-out marks were also observed, indicating that several particles detached from the matrix during a fracture. These features suggest weak filler–matrix interfacial bonding and suboptimal dispersion at this low filler loading, limiting the reinforcing effect of PACP. In the case of PLA with 2 vol% PACP (Figure 9c), the fracture surface showed localised plastic deformation. PACP particles were more uniformly dispersed and often remained embedded within the matrix, even after fracture. Cracks were observed to propagate through some particles rather than around them, indicating enhanced filler–matrix adhesion. These microstructural features are consistent with the relatively improved mechanical properties and elongation at break observed at this composition.

Conversely, the fracture surface of the 3 vol% PACP composite (Figure 9d) revealed a rough, porous morphology with extensive particle pull-out and large voids. The presence of aggregated PACP clusters and interfacial gaps points to poor dispersion and weak interfacial integrity at higher filler loading. These microstructural flaws act as stress risers and explain the notable decline in tensile strength and stiffness at 3 vol%.

## 4. Conclusions

This study explored the fabrication and characterisation of PLA-based biocomposites reinforced with surface-modified pineapple core powder (PACP), produced through extrusion and fused deposition modelling (FDM). By incorporating PACP at low volume fractions (1–3 vol%) and applying silane surface treatment, improved filler dispersion and interfacial compatibility with the PLA matrix were achieved, as confirmed by morphological observations. Rheological testing showed an increase in the melt flow index (MFI), particularly at 2 vol% PACP, indicating better processability. Thermal analyses showed an increase in crystallinity (X_C_) and the emergence of cold crystallisation (T_CC_), demonstrating PACP’s nucleating effect. Although slight reductions in the thermal degradation temperatures (T_5_ and T_max_) were observed with increasing the PACP content, the thermal stability remained sufficient for common thermoplastic processing. Mechanical testing showed that PACP addition improved stiffness in the extruded samples but reduced tensile strength and ductility. For 3D-printed composites, low PACP contents (1–2 vol%) led to modest improvements in elongation at break, likely due to enhanced melt flow and better layer adhesion. However, at 3 vol%, the mechanical properties deteriorated, likely due to filler agglomeration and poor interfacial bonding, as evidenced by fracture morphology. Overall, the 2 vol% PACP formulation was identified as optimal, offering a balanced improvement in printability, crystallinity, and mechanical performance. These findings highlight the potential of PACP as a low-cost, sustainable filler derived from agricultural waste. The PLA/PACP composites developed here show promise for low-load, biodegradable applications such as consumer goods, educational tools, and 3D-printed prototypes. Future research will aim to further enhance interfacial bonding and expand its use in advanced biocomposite systems aligned with circular economy principles.

## Figures and Tables

**Table 2:** Extrusion parameters for PLA/PACP composite filament.

Extruder Zone	Temperature (°C)
Feed zone	160
Compression zone	170
Metering zone	180
Die zone	180
Screw speed	10 rpm

**Table 3:** FDM printing parameters.

Parameter	Setting
Nozzle temperature	205 °C
Nozzle diameter	0.6 mm
Build plate temperature	60 °C
Infill density	100%
Infill pattern	Line +45°/−45°

**Table 4:** Melt flow index of PLA and PLA/PACP composites.

Samples	MFI (g/10 min)
PLA	31.6
PLA/1 vol% PACP	32.1
PLA/2 vol% PACP	35.6
PLA/3 vol% PACP	35.0

**Table 5:** TGA-derived thermal decomposition data for PLA and PLA/PACP composites.

Samples	T_5_ (°C)	T_max_ (°C)	Max. dev. (%/°C)	T_75_ (°C)	Residue (%)
PLA	327.6	362.5	−3.346	366.5	0.14
PLA/1 vol% PACP	322.5	360.0	−3.025	363.4	0.25
PLA/2 vol% PACP	313.8	359.0	−2.674	361.1	0.39
PLA/3 vol% PACP	310.2	358.1	−2.584	360.0	0.59

**Table 6:** Thermal transition parameters from DSC analysis of PLA and PACP-filled PLA composites.

Samples	T_g_ (°C)	T_cc_ (°C)	T_m_ (°C)	X_c_ (%)
PLA	59.66	-	151.74	35.71
PLA/1 vol% PACP	57.13	121.11	151.41	40.27
PLA/2 vol% PACP	59.37	121.13	151.88	47.29
PLA/3 vol% PACP	58.44	121.96	151.46	38.19

**Table 7:** Mechanical properties of extruded PLA and PLA/PACP filaments.

Samples	Ultimate Strength (MPa)	Modulus (MPa)	Elongation at Break (%)
PLA	52.43 ± 2.48	1463.33 ± 5.77	5.58 ± 0.81
PLA/1 vol% PACP	46.78 ± 4.87	1412.50 ± 38.62	4.38 ± 0.66
PLA/2 vol% PACP	46.91 ± 3.13	1518.82 ± 67.63	4.98 ± 0.39
PLA/3 vol% PACP	45.57 ± 3.84	1427.50 ± 56.57	5.05 ± 0.75

**Table 8:** Mechanical properties of 3D-printed PLA and PLA/PACP composites.

Samples	Ultimate Strength (MPa)	Modulus (MPa)	Elongation at Break (%)
PLA	4.52 ± 0.26	267.50 ± 0.71	2.44 ± 0.14
PLA/1 vol% PACP	4.50 ± 0.42	253.50 ± 16.26	2.70 ± 0.08
PLA/2 vol% PACP	4.65 ± 0.56	272.67 ± 17.56	3.00 ± 0.24
PLA/3 vol% PACP	3.82 ± 0.92	223.00 ± 22.63	2.55 ± 0.33

## Data Availability

The data are contained within the article.
